# Performance of CT-guided spatial normalization for semi-quantification of dopamine transporter SPECT and detection of nigrostriatal degeneration

**DOI:** 10.1007/s12149-026-02204-1

**Published:** 2026-04-13

**Authors:** Alae Eddine El Barkaoui, Christian Scheiber, Stephane Thobois, Ralph Buchert, Marc Janier, Thomas Grenier, Anthime Flaus

**Affiliations:** 1 Hospices Civils de Lyon, Département de médecine nucléaire, F-69000 Lyon, France; 2 INSA‐Lyon, Université Claude Bernard Lyon 1, UJM-Saint Etienne, CNRS, Inserm, CREATIS UMR 5220, U1294, F‐69100 Lyon, France; 3 Université Claude Bernard Lyon 1, Institut des Sciences Cognitives Marc Jeannerod, UMR 5229, CNRS,, Centre de Recherche en Neurosciences de Lyon (CRNL), Lyon, France; 4 Université Claude Bernard Lyon 1, Centre de Recherche en Neurosciences de Lyon (CRNL), CNRS UMR 5292, INSERM U1028, Bron, France; 5 Hospices Civils de Lyon, Hôpital Pierre Wertheimer, Centre Expert Parkinson NS-PARK/FCRIN, Département de neurologie, F-69100 Lyon, France; 6 Université Claude Bernard Lyon 1, Faculté de Médecine et de Maïeutique Lyon Sud Charles Mérieux, Oullins, France; 7 Department of Diagnostic and Interventional Radiology and Nuclear Medicine, University Medical Center Hamburg-Eppendorf, Martinistr. 52, 20246 Hamburg, Germany; 8 Université Claude Bernard Lyon 1, Faculté de Médecine Lyon Est, Lyon, France; 9 Laboratoire d’Automatique, de Génie des Procédés et de Génie Pharmaceutique (LAGEPP, UMR 5007, UCBL1 CNRS), Lyon, France

**Keywords:** [^123^I]FP-CIT, DATSCAN, Spatial normalization, Semi-quantitative analysis, Parkinsonian syndromes

## Abstract

**Objective:**

The present study aims to assess the performance of a CT-guided spatial normalization method (CT-method) for the anatomical region-of-interest (ROI)-based semi-quantification of dopamine transporter (DAT) single photon emission computed tomography (SPECT) images and the detection of nigrostriatal degeneration as compared to an effective SPECT template-based method (MSPECT-method) and visual analysis performed by an expert reader.

**Methods:**

Patients who underwent [^123^I]FP-CIT SPECT/CT in the *Hospices Civils de Lyon* between 2008 and 2018 for clinically uncertain parkinsonian syndromes were included. The proposed CT-method aimed to spatially normalize the jointly acquired CT scans and apply the deformation fields to the coregistered SPECT images. It was compared to an effective SPECT template-based method using multiple templates as target for the spatial normalization (MSPECT-method). The distribution of specific binding ratios (SBR) was compared between both methods and the SBR classifications were compared to an expert’s visual classification of the scans, which served as the reference.

**Results:**

Overall, 1156 patients (mean age ± SD = 68.7 ± 11.5; 52.6% male) were included. The CT-method provided a good separation between the normal and reduced SBR, with a higher effect size of the distance between the Gaussians (3.31 vs 3.11) and smaller overlap (6.44% vs 8.96%) compared to the MSPECT-method. Both the CT-method and MSPECT-method demonstrated high classification accuracy (96.7%, 95% CI: 95.7-97.7% vs. 94.6%, 95% CI: 93.3-95.9%), sensitivity (96.0%, CI: 94.3-97.7% vs. 89.7%, CI: 87.1-92.3%), and specificity (97.3%, CI: 96.1-98.6% vs. 98.7%, CI: 97.9-99.6%), respectively.

**Conclusions:**

The proposed CT-guided spatial normalization method for automated semi-quantitative [^123^I]FP-CIT SPECT analysis is a viable option when CT images are available. It offers objective spatial normalization and provides high accuracy for the detection of nigrostriatal degeneration, closely aligning with an expert’s visual interpretation.

**Supplementary Information:**

The online version contains supplementary material available at 10.1007/s12149-026-02204-1.

## Introduction

Single photon emission computed tomography (SPECT) of dopamine transporter (DAT) using the radioligand iodine 123-radiolabeled 2β-carbomethoxy-3β-(4-iodophenyl)-N-(3-fluoropropyl) nortropane ([^123^I]FP-CIT) is used to detect the loss of nigrostriatal dopaminergic neuron terminals in patients with clinically uncertain parkinsonian syndromes [1]. While clinical follow-up criteria combined with visual analysis by an expert reader are the reference for assessing [^123^I]FP-CIT SPECT images in daily clinical practice [2, 3], a semi-quantitative analysis can be used to complement this approach [4]. The most common method for semi-quantification relies on the computation of the specific binding ratios (SBR). Region-of-interest (ROI)-based techniques can be used to assess DAT binding in the specific region (striatum) and non-specific regions (e.g. occipital region) [5]. SBR values obtained from a subject are compared with normative data obtained under the same processing pipelines. This can be useful in different contexts, such as complementing visual analysis in difficult cases [4], providing user-independent evaluation of [^123^I]FP-CIT SPECT images [6], assessing the progression of nigrostriatal degeneration and enabling early disease detection [7], and analyzing large datasets of [^123^I]FP-CIT SPECT images when automated semi-quantification is performed [8, 9].

ROI-based semi-quantification can be based on anatomical ROIs or geometrical ROIs to delineate the region of specific and non-specific uptakes. Geometrical ROIs can be defined large enough to include partial volume counts, thereby reducing the variability in the positioning of the ROIs. However, geometrical ROI-based semi-quantification methods, such as the Southampton quantification method [10], can be sensitive to the reconstruction and calibration strategy [11] and do not allow a separate calculation of the SBR for the caudate and putamen. Conversely, while anatomical ROI-based semi-quantification methods using atlas-based image processing methods can delineate the caudate and putamen separately, most of them require a more accurate spatial normalization to a reference space [12–14]. The standard-of-truth for spatial normalization aims to spatially register a T1-weighted magnetic resonance image (MRI) and then apply the optimal deformation fields to the coregistered functional image [15]. However, since acquisition of [^123^I]FP-CIT SPECT images does not necessarily require additional structural images, most semi-quantitative analyses rely on the use of a single SPECT template representative of a normal level of DAT binding as target for the spatial normalization [16, 17]. However, this approach is sensitive to the DAT density and may be prone to errors when it is low, as spatial normalization, using popular cost-functions, tends to stretch low uptakes to match the uptake in the templates. To overcome this drawback, adaptive registration methods and multiple templates representative of normal and Parkinson-typical reduction of striatal uptake were developed to perform a more accurate spatial normalization [18, 19]. Another approach to overcome the bias related to DAT levels is to use CT images, when these are acquired jointly with the functional images, to assist in warping them [15, 20]. This can be a more appealing approach due to the increased availability of hybrid SPECT/CT and the use of CT [20, 21]. The CT-guided method works independently from the DAT level as it uses only the structural images to generate the deformation fields that can then be applied to the SPECT images.

The aim of the present study was to assess the performance of an automated CT-guided spatial normalization method for the semi-quantification of DAT SPECT images by comparing it to an effective SPECT template-based spatial normalization method. To that end, the SBR distributions were compared between both methods and the SBR classification was then compared to a single expert’s visual analysis, which served as the reference.

## Materials and methods

### Patients

Patients who underwent a [^123^I]FP-CIT SPECT/CT acquisition at the *Hospices Civils de Lyon* nuclear medicine department between 2008 and 2018 to support the diagnosis of clinically uncertain parkinsonian syndromes were reviewed for inclusion. Exclusion criteria were scans with brain injuries, strokes, or tumors (n = 6), severe hydrocephalus (n = 34), no consent to participate (n = 1).

### SPECT/CT imaging

The SPECT/CT scanner (Symbia^®^ T2; Siemens Healthineers, Erlangen, Germany) used integrates low-energy high-resolution collimators. The CT images were acquired in the helical scanning mode with the following parameters: 130 kV, 150 mAs, reconstruction matrix = 512 × 512, voxel size = 0.59 × 0.59 × 1.5 mm. The SPECT images were obtained in circular step and shoot mode acquiring 120 projection angles over 360° (each projection lasted 30 seconds). The SPECT acquisition was held with the following parameters: in-plane pixel size = 3.9 × 3.9 mm, hardware zoom = 1.23 × 1.23, slice thickness = 3.9 mm, reconstruction matrix = 128 × 128, photopeak imaging window = 159 keV ± 8%, and acquisition time = 30 min. The mean injected dose of [^123^I]FP-CIT was 185.97 ± 14.17 MBq (range 158 to 211 MBq), and the image acquisition started 3 hours after the injection. SPECT images were reconstructed using a commercial 3-dimensional (3D) ordered subset expectation-maximization algorithm (Flash3D^®^; Siemens Healthineers) with 10 iterations, 8 subsets. Attenuation correction relied on µ-map derived from the downsampled CT and scatter correction used a triple energy window method. Images were smoothed using a 3D spatial Gaussian filter (full-width-at-half-maximum, 8 mm). The datasets generated and/or analyzed in the present study are not publicly available due to institutional restrictions on patient confidentiality.

### Automated semi-quantitative analysis of [^123^I]FP-CIT SPECT images

#### CT-guided spatial normalization and semi-quantification of [^123^I]FP-CIT SPECT images

To perform the CT-guided spatial normalization of [^123^I]FP-CIT SPECT images, a preliminary step was required to manually correct cases with potential misalignment between images from both modalities. CT images were then stereotactically normalized to a standard space and the optimal generated deformation fields were then applied to the SPECT images using statistical parametric mapping (SPM12, Wellcome Trust Centre for Neuroimaging, London, UK) [22]. In the first step, [^123^I]FP-CIT SPECT and CT images were aligned using the “Coregister” module of SPM12. Visual inspection was subsequently performed as a quality control to confirm the absence of misregistration in all cases following the coregistration step. In the second step, we converted the intensity of CT images to the image brightness range of a CT template [23]. This intensity transformation was intended to increase the intensity range representing the cerebrospinal fluid (CSF) and brain tissue and reduce intensity contrast between the latter and the skull, to improve the elastic non-rigid spatial registration with the CT template. Then, the intensity-transformed CT images were subjected to a non-rigid transformation with the CT template. In the last step, spatially registered CT images were subject to a second non-rigid spatial registration using tissue probability maps (TPMs) from the Multichannel Illinois Institute of Technology & Rush University Aging (MIITRA) atlas [24]. This atlas was specifically designed for studies involving older subjects. The transformations from the two non-rigid spatial registrations were combined and then applied to the coregistered [^123^I]FP-CIT SPECT images. The primary aim of this tuned spatial registration framework was to compensate for potentially enlarged lateral ventricles that may occur with normal aging [25]. After the spatial normalization, pre-defined regions from the automated anatomical labeling atlas (AAL3) in MIITRA space were overlaid on the SPECT images to compute the mean uptake values.

#### SPECT-based spatial normalization and semi-quantification of [^123^I]FP-CIT SPECT images

To perform the SPECT-based spatial normalization we used multiple [^123^I]FP-CIT templates as target for the spatial normalization. These templates, representing different levels of striatal uptake, were the same as those used in the study by Apostolova et al. [18]. In this approach, the spatial normalization algorithm will try to find the best linear combination of the [^123^I]FP-CIT templates to best model the intensities in the [^123^I]FP-CIT images. Spatial normalization of the individual SPECT image from native space to the Montreal Neurological Institute (MNI) space was conducted using affine transformation without non-linear warping using the “Old normalization” module in SPM12. Applying non-linear warping can introduce a bias when comparing subjects with reduced uptakes to those with normal uptakes, as this type of warping preserves the original anatomical structures to a lesser extent and can artificially alter their size and shape [26]. The aim of the proposed approach was to maintain the local intensities and prevent artificial information from altering the outcome of the diagnosis. The affine regularization was set to MNI space, and the spatially normalized images were not modulated in order to preserve the intensities of the original images. SPECT images were then sampled using trilinear interpolation, and the voxel size was 2 × 2 × 2 mm. The putamen and occipital lobe ROIs of the AAL3 atlas were used for the computation of the regional uptakes [27].

For the sake of simplicity, we will refer to the CT-guided normalization of [^123^I]FP-CIT SPECT images as the CT method and to the multiple [^123^I]FP-CIT SPECT templates approach as the MSPECT method. Diagrams of both methods are given in Online Resource 1.

### Semi-quantitative analysis

#### The used reference for [^123^I]FP-CIT SPECT classification

The SBR classifications obtained from both semi-quantification methods were compared to a visual analysis performed by a single expert reader (15 years of experience). While the clinical diagnosis after follow-up is the gold standard for parkinsonian syndromes, the aim of this study was to assess the performances of automated image analysis pipelines by comparing them to the routine clinical interpretation of the scans. Therefore, the single expert’s visual classification of the scans as normal or abnormal was used as the only reference for the present study.

#### SBR computation

The semi-quantitative analysis of [^123^I]FP-CIT SPECT images used herein relied on the positioning of predefined putamen and occipital cortex ROIs in normalized space [1]. The putamen was chosen as the specific uptake region in order to increase the sensitivity of the semi-quantification as the Parkinson-typical reduction of striatal uptake predominantly begins in the putamen [28, 29]. SBR values using both the CT method and the MSPECT method were calculated according to the following formula [5]:

1 $$\mathrm{SBR}=\frac{\text{Mean count in the putamen ROI }-\text{ Mean count in the occipital ROI}}{\text{Mean count in the occipital ROI}}$$

The number of voxels within the ROIs was fixed and did not take into consideration potential volumetric decrease due to aging [30]. The minimum SBR value between the two hemispheres was used for the semi-quantitative analysis as it is more relevant for classification than the mean SBR value [18].

#### SBR analysis

The SBR distribution and classification obtained from both semi-quantification methods were then analyzed. Firstly, we characterized the distribution of the minimal putaminal SBR by fitting a Gaussian mixture model (GMM) to estimate the separation between normal and reduced SBR. Each Gaussian component corresponds to a distinct subpopulation within the data, which, in our study, are the normal and reduced SBR groups. The process involves an expectation-maximization algorithm to iteratively estimate the parameters of each Gaussian component. These parameters include the mean (the central value of the SBR for each group), the standard deviation (SD, the spread or variability of SBR within each group), and the mixing proportion (the proportion of data points belonging to each group). By fitting the GMM, we can objectively model the underlying distributions without making prior assumptions about a single cutoff point. This approach is superior to using a simple threshold because it accounts for the natural variability and overlap between the two subpopulations. The separation between the normal and reduced SBR was then quantified using two key metrics derived from the GMM parameters. The first metric was the effect size of the distance between the Gaussians, quantifying the difference between the means of the two components in terms of their pooled SD. It is a standardized measure, making it easier to compare effect sizes across different datasets. The formula used was:

2 $$\text{effect size}= \frac{{\mathrm{M}}_{2}-{\mathrm{M}}_{1}}{\surd \frac{{\mathrm{SD}}_{1}^{2}+{\mathrm{SD}}_{2}^{2}}{2}}$$

Where M1 and M2 were the mean values, SD1, and SD2 were the SD of the Gaussian functions. The second computed metric was the degree of overlap between the components of the GMM fits. A value closer to 0% means less overlap (better separation), and a value closer to 100% means more overlap. The formula used for the overlap was:

3 $$\mathrm{overlap}={\mathrm{e}}^{-\frac{{(\mathrm{M}1-\mathrm{M}2)}^{2}}{2({\mathrm{SD}1}^{2}{+\mathrm{SD}2}^{2})}}$$

This methodology allows us to provide a data-driven, quantitative measure of the separation performance of each semi-quantification method, directly addressing the core objective of our study without being affected by the limitations of the visual analysis by a single expert, which was used as reference.

Secondly, we performed a classification analysis of SBR values by comparing them to the results obtained from a single expert’s visual analysis using the cutoff maximizing accuracy to obtain the following standard performance metrics: accuracy, sensitivity, specificity, and area under the curve (AUC).

### Statistical analysis

Analyses were conducted using MATLAB (R2021a; The MathWorks Inc., Natick, MA, USA). All p-values were given two-sided. Statistical significance was defined as p < 0.05. Descriptive statistics were presented as follows: continuous variables as means and SD or medians and interquartile range (IQR) and range, and compared using the Wilcoxon signed rank test or the Student’s t-test, according to their distribution. The degree of concordance between the two SBR measurements from both methods was assessed using intra-class correlation coefficient and Bland-Altman plots. The degree of classification agreement between both semi-quantitative methods was assessed using the Cohen’s kappa coefficient. The 95% confidence intervals (95% CI) were estimated for all diagnostic performance metrics.

## Results

### Patient data

In total, 1197 patients underwent a [^123^I]FP-CIT SPECT/CT acquisition over the study period; 41 patients (3.43%) were excluded. Overall, images from 1156 patients were finally included (mean age ± SD = 68.7 ± 11.5; 52.6% male). Of note, regarding the CT method, preliminary reorientation before spatial normalization was required in 8 cases (0.69% of cases), where a significant misalignment was noticed.

### SBR distributions using CT and MSPECT methods

**Fig 1:**
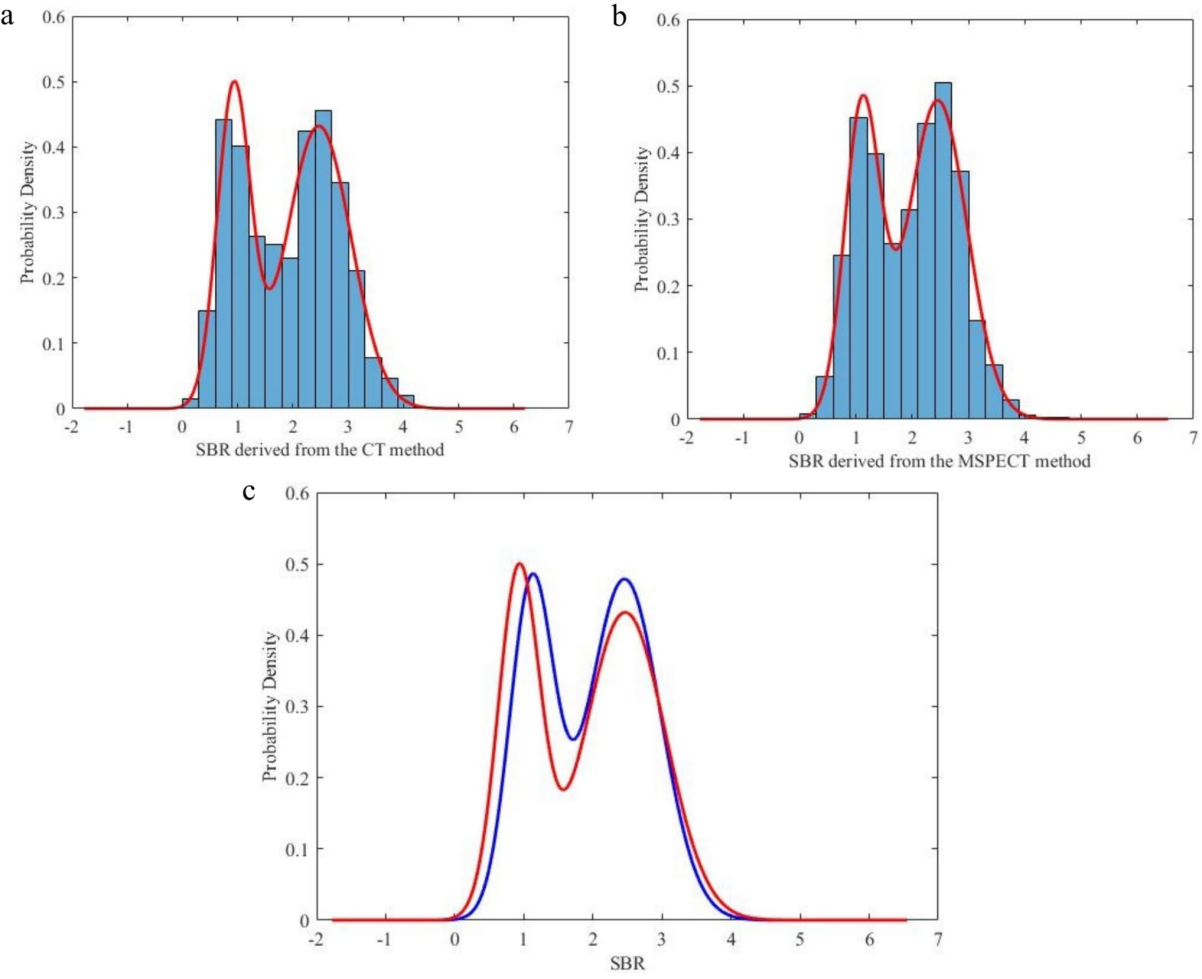
Specific binding ratios (SBR) distribution for both methods. a: The fitted Gaussian mixture model (GMM) of SBR derived from the CT-guided normalization of [^123^I]FP-CIT SPECT images method (CT method) and b: the multiple [^123^I]FP-CIT SPECT templates method (MSPECT method). c: Superposition of the GMM fits of SBR derived from the CT method (red) and the MSPECT method (blue)

The fitted GMM derived from the CT and MSPECT methods revealed that the CT method provided a higher effect size (3.31) and a lower overlap (6.44%) of the two Gaussians representative of the putaminal SBR compared to the MSPECT method (effect size = 3.11, overlap = 8.96%; Fig. 1). The cutoff derived from the fitted GMM of the CT method (cutoff = 1.70) was smaller than that of the MSPECT method (cutoff = 1.79).

**Fig 2:**
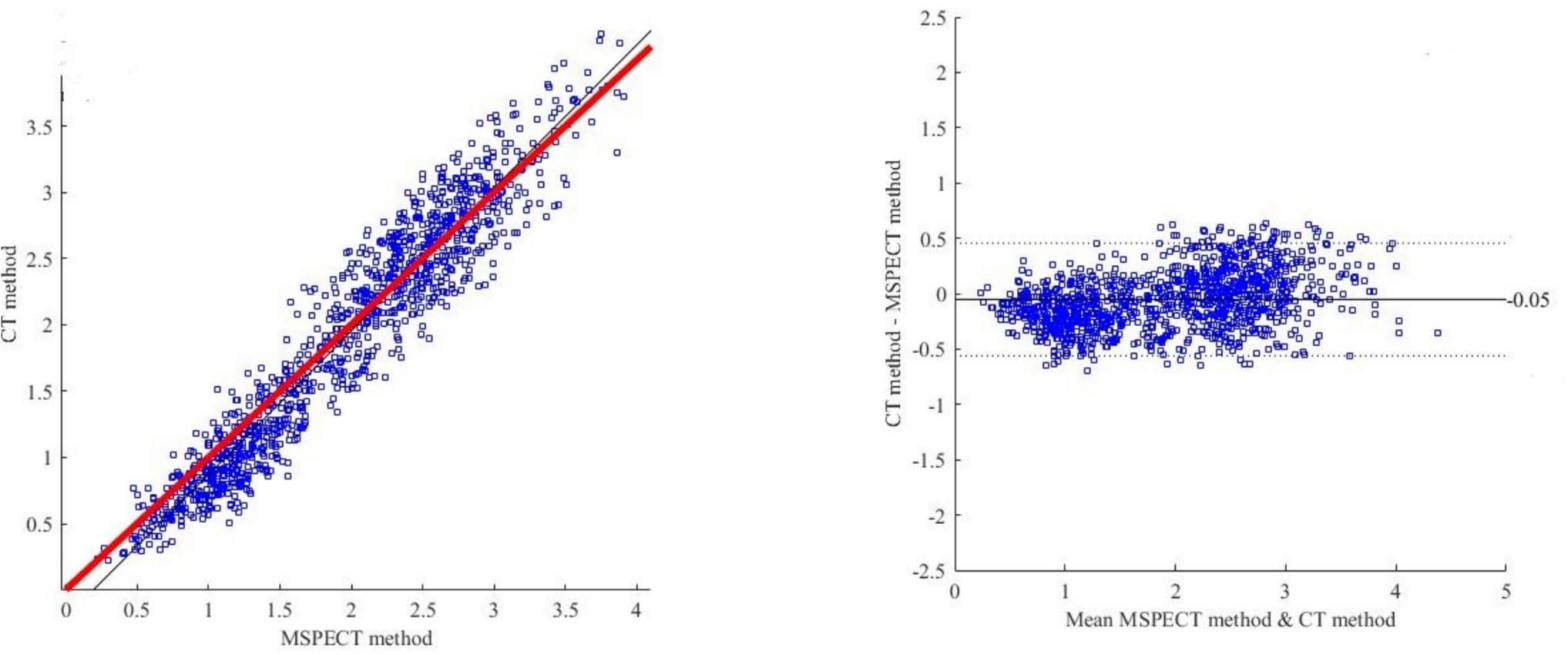
Intra-class correlation scatter (left) and Bland-Altman plot (right) assessing agreement between measurements of specific binding ratios (SBR, blue circles) derived from the CT-guided normalization of [^123^I]FP-CIT SPECT images method (CT method) and the multiple [^123^I]FP-CIT SPECT templates method (MSPECT method). The red line indicates perfect agreement between both methods while the black line indicates the actual agreement

For the CT method, the fitted GMM resulted in an SD of 0.30 for the first component and 0.58 for the second component. The mixing proportions were 0.37 and 0.63 for the first and second components, respectively. For the MSPECT method, the fitted GMM resulted in an SD of 0.32 for the first component and 0.52 for the second component. The mixing proportions of the MSPECT method were equal to those of the CT method (0.37 and 0.63 for the first and second components, respectively). The SBR values derived from both methods were well correlated (p < 0.001; r^2^ = 0.92; Fig. 2). The mean difference between the SBR computed from both methods was 0.05 (Fig. 2)

The mean putaminal SBR derived from the CT method was 2.03 (IQR, 1.05 to 2.63, range, 0.22 to 4.20) and that derived from the MSPECT method was 2.04 (IQR, 1.23 to 2.58, range, 0.22 to 4.55). For patients with normal striatal uptake, the mean SBR computed by the CT method was 2.61 (IQR, 2.27 to 2.91, range 1.51 to 4.20) whereas that computed by the MSPECT method was 2.57 (IQR, 2.28 to 2.82, range, 1.50 to 4.55). For patients with reduced striatal uptake, the mean SBR computed by the CT method was 1.05 (IQR, 0.76 to 1.29, range, 0.22 to 2.28) whereas that computed by the MSPECT method was 1.22 (IQR, 0.97 to 1.47, range, 0.22 to 2.24)

### Classification performance assessment: comparison with visual analysis

According to the visual analysis of [^123^I]FP-CIT SPECT images, 632 (54.67%) were considered as normal and 524 (45.33%) as pathological. The best cutoffs for maximizing accuracy were 1.755 for the CT method and 1.710 for the MSPECT method. The classification accuracy of the CT method was slightly superior to that of the MSPECT method (96.7%, 95% CI: 95.7-97.7% vs 94.6%, 95% CI: 93.3-95.9%, p = 0.0018). The classification sensitivity was also higher with the CT method compared to the MSPECT method (96%, CI: 94.3-97.7% vs 89.7%, CI: 87.1-92.3%, p < 0.001). In contrast, the classification specificity of the MSPECT method was higher than that of the CT method (98.7%, CI: 97.9-99.6% vs 97.3%, CI: 96.1-98.6%, p = 0.049; Table 1). The Cohen’s kappa coefficient (k = 0.95) revealed an almost perfect classification agreement between both methods. The AUC of the CT and MSPECT methods were 0.9952 and 0.9903, respectively (p = 0.064).

**Table 1:** Confusion matrix as well as accuracy, sensitivity, and specificity according to the cutoff that gave maximum accuracy for both the CT-guided normalization of [^123^I]FP-CIT SPECT images method (CT method) and the multiple [^123^I]FP-CIT SPECT templates method (MSPECT method)

	Cutoff	Accuracy	Sensitivity	Specificity	TN	TP	FP	FN
CT method	1.755	96.7%	96%	97.3%	615	503	17	21
MSPECT method	1.710	94.6%	89.7%	98.7%	624	470	8	54

TN: True negative; TP: True positive; FP: False positive; FN: False negative

**Fig 3:**
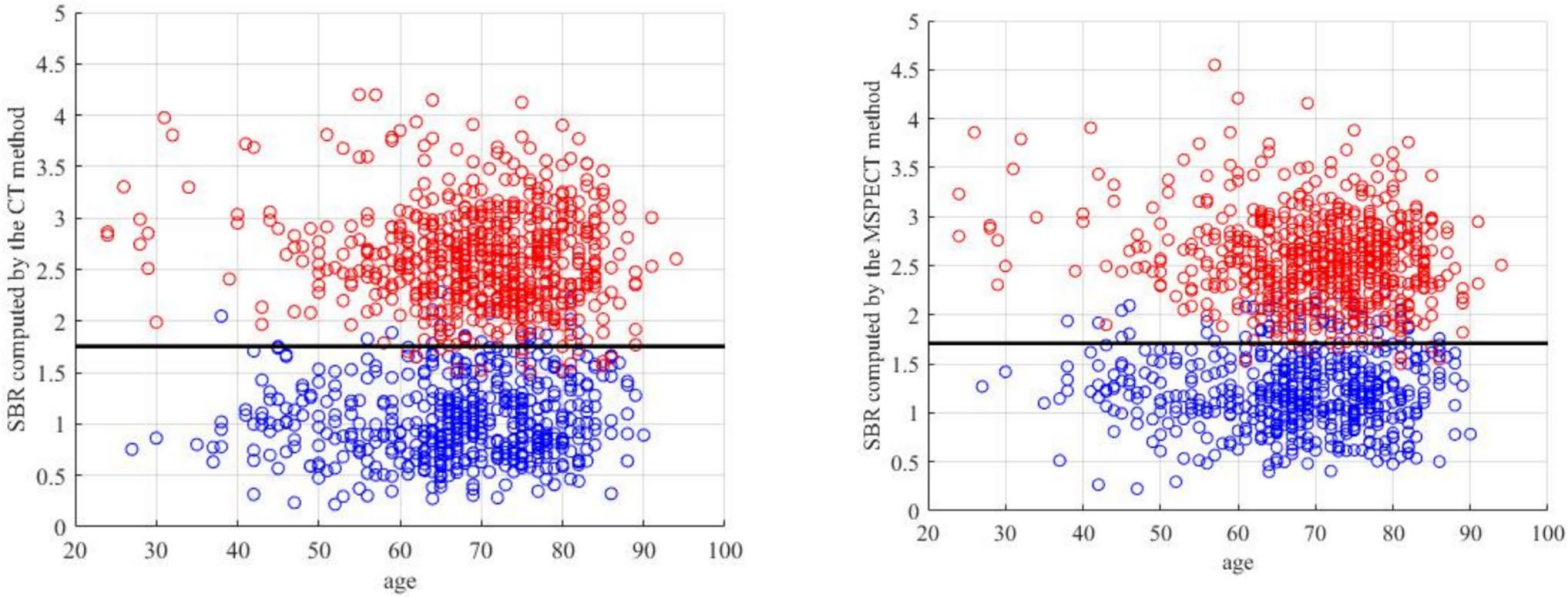
Scatter plots of putamen specific binding ratios (SBR) computed by the CT-guided normalization of [^123^I]FP-CIT SPECT images method (CT method) and the multiple [^123^I]FP-CIT SPECT templates method (MSPECT method) according to age. SBR classified as normal by the visual analysis are indicated by red dots, those classified as pathological by the visual analysis are indicated by blue dots

The scatter plots of SBR according to age showed a slight improvement of the separation between normal and reduced uptake cases according to visual analysis when using the CT method compared to the MSPECT method (Fig. 3).

There was no statistically significant difference in the mean difference of the false positive SBR values from the best cutoffs when comparing the two methods (p = 0.8; Table 2). Conversely, the mean difference of false negative SBR values from the cutoff was lower for the CT method than for the MSPECT method (p = 0.21; Table 2).

**Table 2:** Specific binding ratios (SBR) of false negative (FN) and false positive (FP) cases obtained from both the CT-guided normalization of [^123^I]FP-CIT SPECT images method (CT method) and the multiple [^123^I]FP-CIT SPECT templates method (MSPECT method) as well as their mean differences from the cutoffs

	Cases	Mean SBR	Mean difference from cutoff (SBR-cutoff)
CT method	FN	1.96	0.20
	FP	1.64	-0.11
MSPECT method	FN	1.96	0.25
	FP	1.60	-0.11

FP: False positive; FN: False negative

## Discussion

The key outcome of the present study is that CT-guided spatial normalization was effective for the semi-quantification of [^123^I]FP-CIT SPECT images and was comparable to the most accurate SPECT-guided method for detecting putaminal dopaminergic deficits. When considering the cutoff that gives the highest accuracy, the CT method resulted in a slightly more accurate and more sensitive classification of [^123^I]FP-CIT SPECT images.

The findings of the present study on the performance of CT-guided semi-quantification of functional images are consistent with previous reports [15, 21, 31]. Specifically, previous studies [15, 31] showed that the ROI delineation from CT-guided methods was highly correlated with the widely accepted and accurate MRI-guided methods and were highly effective in semi-quantification of functional images. CT-guided methods can therefore be used to delineate ROIs on the coregistered [^123^I]FP-CIT images since hybrid SPECT/CT systems allow sequential acquisition of SPECT and CT in a single study.

The proposed CT-guided method works independently from the uptake level and its performance is therefore expected to be superior to widely used SPECT-guided methods that usually use one single SPECT template with normal uptake as target for the warping. This is because popular algorithms for spatial normalization, such as least squares, work by minimizing the sum of the squared differences between the image that is to be normalized and the template. In cases with a lack of DAT binding in some regions such as the anterior putamen, the spatial normalization will tend to stretch the uptake to fill the regions with missing binding, thereby potentially decreasing the accuracy of discriminating between patients with Parkinson’s disease and healthy controls [15]. The MSPECT method helps to overcome this drawback by using multiple SPECT templates with different levels of striatal uptake, which might improve the semi-quantitative analysis of [^123^I]FP-CIT [18]. Interestingly, the accuracy of the classification outcome derived from the CT method was slightly higher than that of the MSPECT method herein. For patients with reduced putamen uptake, the mean putaminal SBR was lower using the CT method; this is likely explained by the fact that the CT and MSPECT methods diverge in their approach to spatial normalization when DAT binding is low. The MSPECT method uses multiple SPECT templates, including those with reduced striatal uptake. While this improves on single-template methods, the spatial normalization algorithm still seeks to match the patient’s SPECT signal with a template, which can lead to a slight overestimation of SBR in patients with severe degeneration. In contrast, the CT method relies solely on the patient’s structural CT scan for spatial normalization. This difference in methodology leads to a more accurate, and thus lower, mean SBR value for the reduced SBR group when using the CT method, thereby more closely reflecting the true extent of nigrostriatal degeneration according to the obtained results. The observed differences in specificity, sensitivity, and false-negative rates are also a direct result of the methods’ underlying principles. MSPECT’s higher specificity and more false negatives are explained by the MSPECT method’s reliance on SPECT templates, which may have higher uptake in some regions, potentially leading to a slight overestimation of the SBR, particularly in the lower range of normal values. This can push some borderline reduced cases into the normal category, resulting in more false negatives and a trade-off that favors a higher specificity (correctly identifying more normal cases). The higher mean SBR for the reduced group and the higher cutoff for the MSPECT method support this conclusion. However, the improvement in accuracy observed using the CT method remains only slight, likely due to the high symptom threshold of neurodegenerative parkinsonism [32], which is well over the between-subject variance induced by the spatial normalization method. It is worth noting that this high symptom threshold was reported to also be larger than the proportion of between-subject variance caused by age and sex in older subjects [33], which is why no age correction was applied in the present study.

Some limitations of the present study must be acknowledged. First, the classification performance was assessed using visual analysis by a single expert reader as reference and the intra-reader agreement was not provided, which may introduce a potential bias. Visual interpretation inherently involves subjectivity [6], the precise impact of which is still debated. While a recent study reported very high inter- and intra-reader agreements (e.g., mean inter-reader kappa of 0.947 (± 0.008) and mean intra-reader kappa of 0.956 (± 0.010) for experienced readers [34]), other studies have highlighted more variable interobserver reproducibility [35]. A possible reason for this discrepancy is that subjectivity becomes more challenging in borderline cases. This is particularly true when analyzing scans from older individuals, where age-related decline in striatal binding [36] can mimic early parkinsonism. Given that these more ambiguous cases are more likely to be influenced by reader-specific thresholds, the use of a single reader as reference herein, rather than a consensus panel [4], could limit the generalizability of the performance metrics obtained. However, a major part of the analyses in the present study did not rely on visual analysis as reference: the binary categorization performance was assessed through effect size and Gaussian overlap analysis, providing an objective, data-driven measure of group separation that is independent of the visual reference. Further studies with more readers are needed to validate the robustness of the proposed method against inter- and intra-reader variabilities. Another limitation is that the performance of both methods was not assessed in cases with atypical striatal uptake reduction patterns (e.g., pronounced reduction in the caudate nucleus uptake with normal putamen uptake, or complete lack of striatal uptake in one hemisphere). This is due to the fact that the binary categorization of [^123^I]FP-CIT SPECT herein was based on the lowest DAT uptake in the putamen between both hemispheres. However, unlike the MSPECT method that uses SPECT templates representative of Parkinson-typical striatal uptake reduction, the CT method is agnostic to the uptake of [^123^I]FP-CIT; its classification accuracy should thus not be impacted by atypical uptakes when computing SBR in specific striatal subregions. Furthermore, cases with brain lesions or hydrocephalus were excluded from the study because both automated spatial normalization algorithms used herein perform poorly in those cases. An alternative approach to deal with lesions and abnormal anatomical landmarks is to use lesion cost-function masking, which requires identifying abnormal regions so that they do not contribute to the normalization transforms [23]. In patients with strongly widened CSF or brain lesions, an alternative semi-quantification approach can be used, in which a wider brain region can be used as reference region in addition to ROI enlargement and intensity scaling in order to reduce the contribution of CSF and brain lesions [37]. However, these alternative approaches require additional image processing steps, which may render the automation and generalization of the processing pipelines more laborious. More advanced tools can be used in similar challenging spatial normalization cases, such as in atrophied brains [38]. An additional limitation relates to the possible misalignment between the CT and the SPECT scans: although we performed a visual check of the coregistration outcome, this represents a drawback of the CT method as it requires additional effort to check the coregistered images for each subject. Nonetheless, the CT method has several advantages and can be useful for different functional imaging modalities and with various radiotracers whose uptakes are restricted to specific brain regions. Moreover, several studies have suggested the feasibility of low-dose CT guided spatial normalization of functional images, by showing, for instance, a high level of concordance between low-dose CT and the gold standard MRI-guided normalization in terms of PET image quantification [31, 39]. This suggests that the fundamental anatomical structures needed for accurate warping remain usable at lower radiation doses, but further studies are needed, using an MRI-guided approach as the gold standard, to evaluate whether it yields the same spatial normalization performance. The CT-guided approach also offers a sufficiently accurate alternative when a prior MRI is not easily accessible and only when a single, streamlined SPECT/CT acquisition is performed. The proposed automated pipeline can aid clinicians in the evaluation of [^123^I]FP-CIT SPECT scans and was built exclusively using widely available and validated tools, and does not require any tracer-specific template to conduct the spatial normalization.

## Conclusions

The proposed CT-guided spatial normalization method for automated semi-quantitative [^123^I]FP-CIT SPECT analysis is a viable option when CT images are available. It offers objective spatial normalization and provides high accuracy for the detection of nigrostriatal degeneration, closely aligning with an expert’s visual interpretation.

## Supplementary Information

Below is the link to the electronic supplementary material.

Supplementary file.1

## Data Availability

The datasets generated and/or analyzed during the current study are not publicly available due to institutional restrictions on patient confidentiality, prior consent, and privacy.

## Abbreviations

3D: 3-Dimensional
AAL: Automated anatomical labeling
AUC: Area under the curve
CSF: Cerebrospinal fluid
CT: Computed tomography
DAT: Dopamine transporter
FN: False negative
FP: False positive
GMM: Gaussian mixture model
[123I]FP-CIT: Iodine 123-radiolabeled 2β-carbomethoxy-3β-(4-iodophenyl)-N-(3-fluoropropyl) nortropane
IQR: Interquartile range
MIITRA: Multichannel illinois institute of technology & rush university aging
MNI: Montreal neurological institute
MRI: Magnetic resonance image
MSPECT: Multiple SPECT templates
PET: Positron emission tomography
ROI: Region-of-interest
SBR: Specific binding ratios
SD: Standard deviation
SPECT: Single photon emission computed tomography
SPM: Statistical parametric mapping
TN: True negative
TP: True positive
TPM: Tissue probability maps
