# Supplementary material for: Performance of CT-guided spatial normalization for semi-quantification of dopamine transporter SPECT and detection of nigrostriatal degeneration
Source: Ann Nucl Med. 2026 Apr 13;40(8):926–36. doi: 10.1007/s12149-026-02204-1 (PMC13388770; doi:10.1007/s12149-026-02204-1)
Supplement: Supplementary file 1 — Supplementary file.1 [file 12149_2026_2204_MOESM1_ESM.docx]

Online resource 1: Diagrams of the CT and MSPECT methods


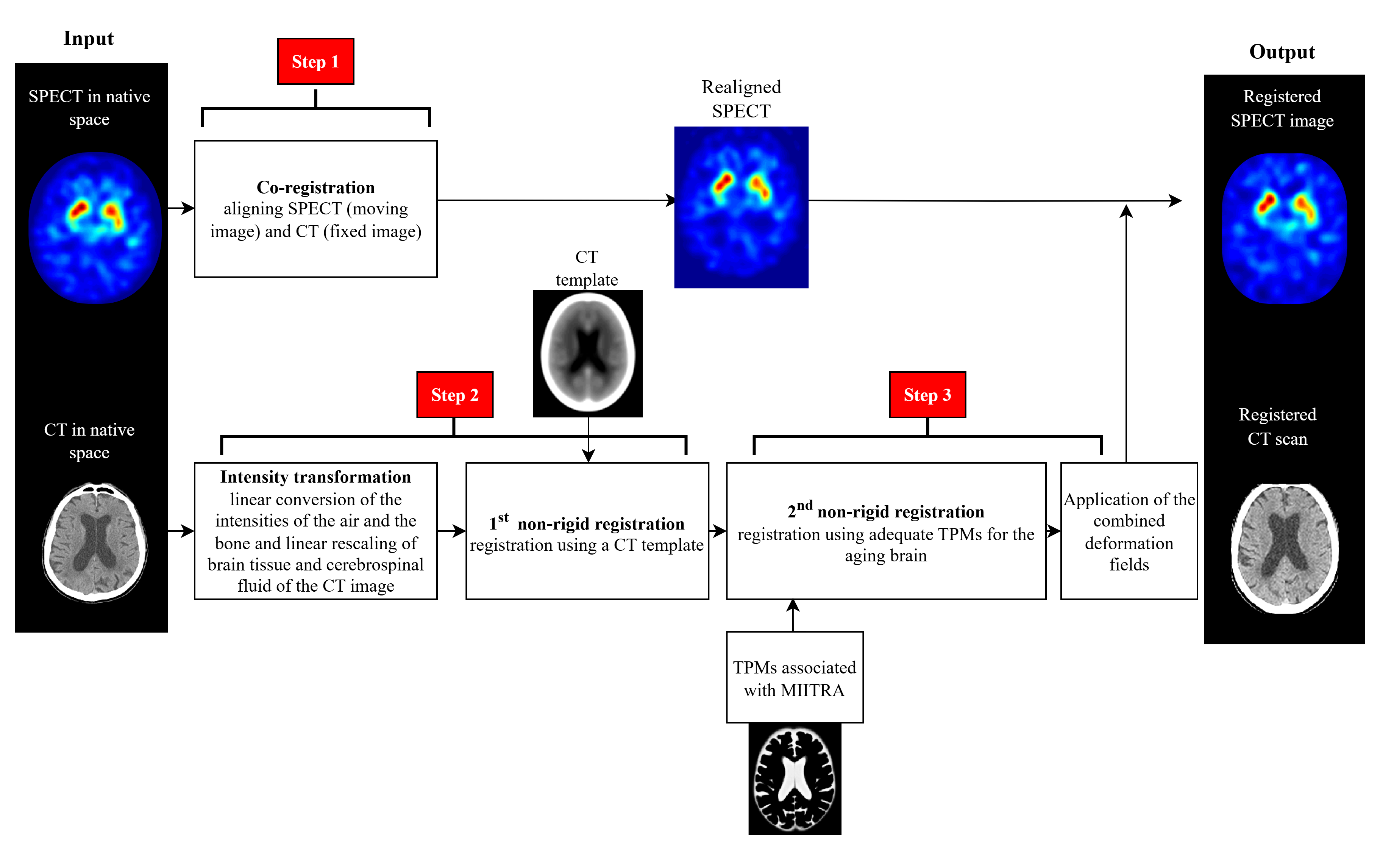
CT-guided spatial normalization pipeline of [^123^I]FP-CIT images (CT method, [1]):


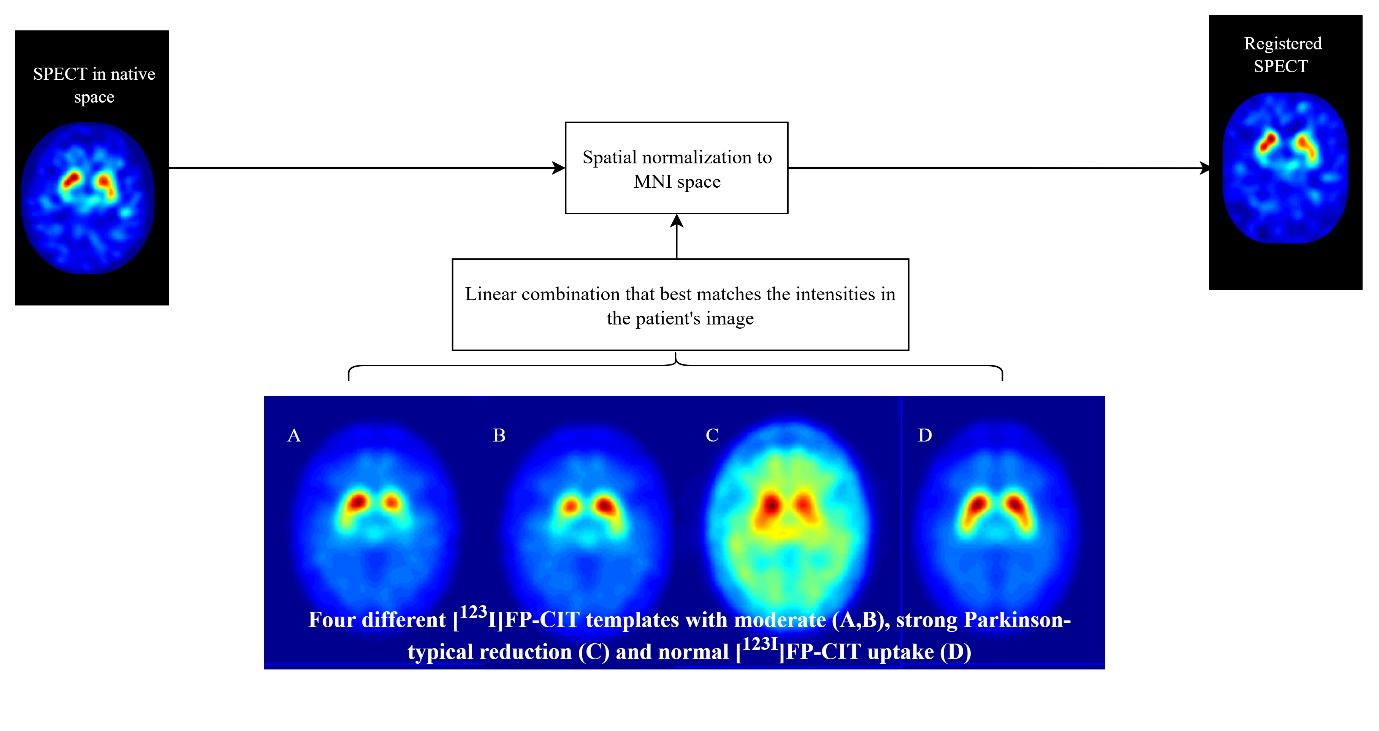
Pipeline of the spatial normalization with multiple [^123^I]FP-CIT templates images (MSPECT method, [2]:

1. El Barkaoui AE, Scheiber C, Grenier T, Janier M, Flaus A. CT-guided spatial normalization of nuclear hybrid imaging adapted to enlarged ventricles: Impact on striatal uptake quantification. NeuroImage. 2024;294:120631. Available from:

2. Apostolova I, Schiebler T, Lange C, Mathies FL, Lehnert W, Klutmann S, et al. Stereotactical normalization with multiple templates representative of normal and Parkinson-typical reduction of striatal uptake improves the discriminative power of automatic semi-quantitative analysis in dopamine transporter SPECT. EJNMMI Phys. 2023;10:25.
